# Supplementary material for: Splice-Junction-Based Mapping of Alternative Isoforms in the Human Proteome
Source: Cell Rep. Author manuscript; Available in PMC 2020 Jan 15. (PMC6961840; doi:10.1016/j.celrep.2019.11.026)
Supplement: 2 [file NIHMS1546469-supplement-2.zip › DF1/PXD009737/TestisLysC/TestisLysC_14_KIAA1210_SLTATQVEPKEEEPNLPLVSEEEK.pdf]

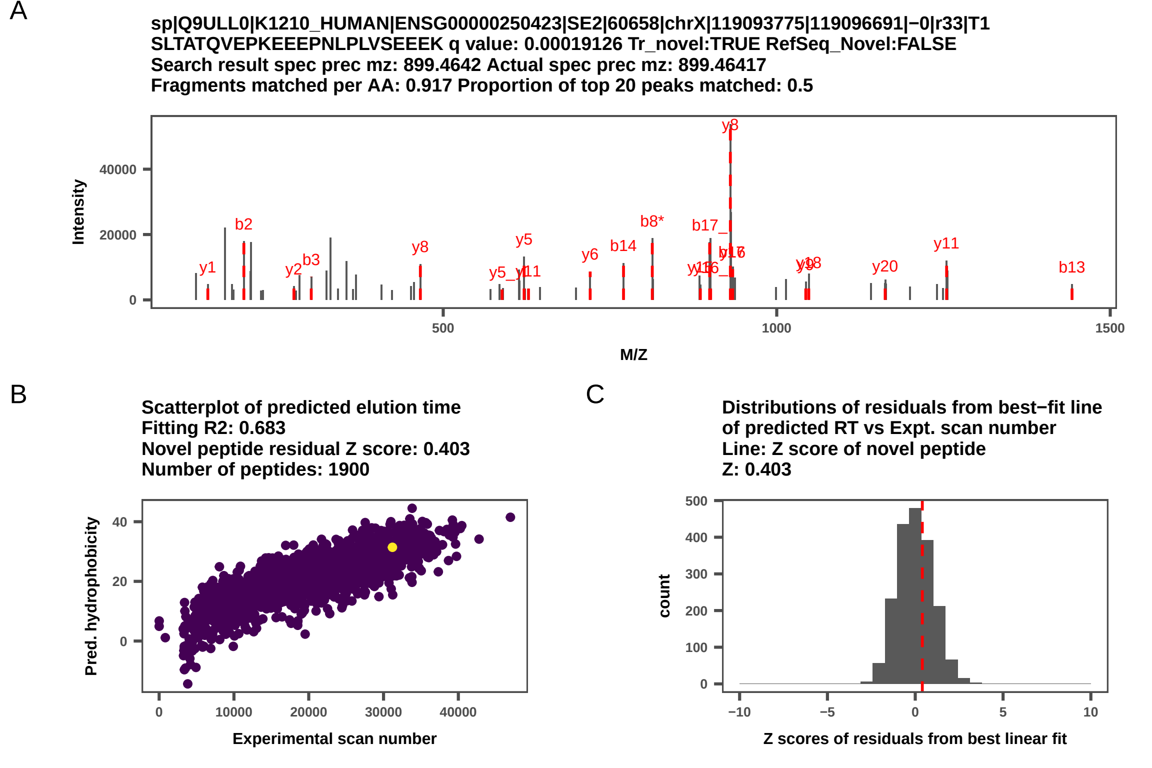
A
sp|Q9ULL0|K1210_HUMAN|ENSG00000250423|SE2|60658|chrX|119093775|119096691|−0|r33|T1
SLTATQVEPKEEEPNLPLVSEEEK q value: 0.00019126 Tr_novel:TRUE RefSeq_Novel:FALSE
Search result spec prec mz: 899.4642 Actual spec prec mz: 899.46417
Fragments matched per AA: 0.917 Proportion of top 20 peaks matched: 0.5
Intensity
M/Z
B
Scatterplot of predicted elution time
Fitting R2: 0.683
Novel peptide residual Z score: 0.403
Number of peptides: 1900
Pred. hydrophobicity
Experimental scan number
C
Distributions of residuals from best−fit line
of predicted RT vs Expt. scan number
Line: Z score of novel peptide
Z: 0.403
count
Z scores of residuals from best linear fit
